# Utilizing Tea Plant Synomones to Attract *Encarsia smithi* for Suppressing *Aleurocanthus spiniferus* in Tea Plantations

**DOI:** 10.3390/plants15030491

**Published:** 2026-02-05

**Authors:** Yiqi Wu, Shanjie Han, Peizhen Fan, Huoxiang Ye, Yanjun Cheng, Yue’er Liang, Xinqiang Zheng, Jianliang Lu, Baoyu Han

**Affiliations:** 1 Tea Research Institute, Zhejiang University, Hangzhou 310058, China; yiqiwu@zju.edu.cn (Y.W.); xqzheng@zju.edu.cn (X.Z.); 2 College of Management Science and Engineering, China Jiliang University, Hangzhou 310018, China; hanshanjie@cjlu.edu.cn; 3 Lu’an Vocational and Technical College, Lu’an 237158, China; fpz@lvtc.edu.cn; 4 Songyang County Agricultural and Rural Bureau of Zhejiang Province, Songyang 323400, China; syyhx7539@163.com; 5 College of Life Science, China Jiliang University, Hangzhou 310018, China; s23090710003@cjlu.edu.cn (Y.C.); s23090710023@cjlu.edu.cn (Y.L.); 6 Hangzhou Tea & Chrysanthemum Technology, Co., Ltd., Hangzhou 310018, China

**Keywords:** citrus spiny whitefly, *Encarsia smithi*, whitefly-pierced leaves, adjacent intact leaves, signaling among plants

## Abstract

The citrus spiny whitefly, *Aleurocanthus spiniferus* Quaintance (Hemiptera: Aleyrodidae), is an important pest of tea, *Camellia sinensis* (L.) Kuntze (Theales: Theaceae). Parasitic wasp, *Encarsia smithi* Silvestri (Hymenoptera: Aphelinidae), is one of the dominant natural enemies of the whitefly. Generally, the whitefly produces four generations per year in Chinese tea plant growing areas. The wasp adult stages are basically synchronized with the nymphal stages of the whitefly. In an indoor Y-tube olfactometer bioassay, odors from both whitefly-pierced tea leaves and adjacent intact tea leaves significantly attracted the wasps, with elevated amounts of *trans*-2-hexenal and methyl salicylate (MeSA) detected from these two types of tea leaves. A four-arm olfactometer bioassay verified that these two compounds and their binary blends significantly attracted the wasps. Bud green sticky boards baited with *trans*-2-hexenal (10^−2^ g mL^−1^), MeSA (10^−2^ g mL^−1^), and five blends of *trans*-2-hexenal and MeSA (1:1, 2:1, 3:1, 4:1 and 5:1, respectively, *v*/*v*) at 10^−2^ g mL^−1^ in hexane solutions captured significantly more wasps than did the un-baited boards, with the 3:1 blend catching the highest number of wasps. To enhance whitefly parasitism by the wasps, from early April to early August, the Attractant 2 lures (each holding a total mass of 80 mg of the 3:1 blend) were hung on tea branches and refreshed every 30 days. Deployment of the controlled release synomone-based attractant lures resulted in 2–3 fold higher parasitism rates by the wasps in the treated plots/sections than those in the CK plots/sections during both the peak periods of whitefly pupae of generation 2 from late July to early August and generation 3 in late August. This study demonstrated that herbivore-induced tea volatiles can be formulated as a synomone-based lure for controlling the whitefly through attracting *E. smithi* in tea plantations.

## 1. Introduction

The citrus spiny whitefly, *Aleurocanthus spiniferus* Quaintance (Hemiptera: Aleyrodidae), is a pest of tea *Camellia sinensis* (L.) Kuntze (Theales: Theaceae) plants and orange (Sapindales: Rutaceae) trees. It reproduces four generations per year in most Chinese tea plant-growing areas. Its nymphal stages of generations 1, 2, 3, and 4 emerge from mid-April to late June, late June to early August, mid-August to mid-October, and mid-October to next late March, respectively. The number of nymphs and pupae inhabiting the underside of leaves in the middle and lower layers of tea bushes accounts for approximately 90% of its total population [1,2]. Both nymphs and adults pierce the tea leaves and suck leaf sap and excrete “honeydew” to breed coal dust fungi, which causes heavy tea sooty mold (*Neocapnodium theae* Hara) development that blackens tea canopies [1,2,3]. The whitefly is very difficult to control, due to their small body size and being covered with wax, their large abundance and overlapping generations. Trapping the mated adults of overwintering generation from end March to early April with attractant-baited sticky boards is considered as one of the most effective tactics for its suppression [1,2,4]. Several tea plant volatile compounds could act as attractants [2,4,5]. On the other hand, herbivore-induced plant volatiles (HIPVs) are specific volatile organic compounds produced by plants in response to herbivory and play a significant role in the attraction of natural enemies and plant defense against herbivore attacks, e.g., methyl salicylate (MeSA) [6]. Traps baited with MeSA were suitable for reducing *Frankliniella occidentalis* (Pergande) (Thysanoptera: Thripidae) in susceptible crops by attracting natural enemies to crop production fields as a part of IPM-based management practices [7]. At the same time, plants can detect herbivore-induced plant volatiles (HIPVs) from their damaged neighbors via stomata and respond by enhancing or priming their defenses against future herbivore attacks [8].

We discovered that the parasitoid wasp, *Encarsia smithi* Silvestri (Hymenoptera: Aphelinidae) parasitizes nymphs (and some pupae) of the whitefly [3,4]. Together with a few other species of parasitic wasps, its parasitism rate on the whitefly usually reaches 30% within tea and forest intercropping tea plantations in the mountainous area of southern Anhui province, with the highest record of 75% [3]. The parasitic wasp population boomed in both July (pupal stage of the 2nd generation of *A. spiniferus*) and August (pupal stage of the 3rd generation of *A. spiniferus*) [1,9]. It is also an exclusive and dominant parasitic wasp of *Aleurocanthus spiniferus* Quaintance in Japanese orange plantations and *Aleurocanthus camelliae* (Kanmiya & Kasai) (Hemiptera: Aleyrodidae) in tea plantations in Japan [10]. According to reference [11], it was imported from China in 1925 and has been used to control *A. spiniferus* populations in tea plantations [12]. Furthermore, recent research disclosed the wasps rapidly spread to the region where the whitefles expanded their distribution and controlled population outbreaks in many sites. However, at some sites where the population density of the whiteflies increased rapidly, and the parasitism rates of *E. smithi* tended to remain at low levels or declined. The range of parasitism rates in 27 tea plantation sites in Shizuoka Prefecture of Japan from 2011 to 2013 was from 0 to 100% with typical parasitism rates being 20% to 60%. The research still verified that parasitism by *E. smithi* could be used as an effective biocontrol measure for controlling *A. camelliae* populations in tea plantations [13]. During 1974–1998, *E. smithi* was introduced from Japan to Hawaii to control the spiny whitefly *Aleurocanthus spiniferus* Quaintance [14]; however, low host density limited the parasitoid establishment. These examples highlight the potential of *E. smithi* as a classical and augmentative biological control agent. A viable question might be “can we enhance the potential of *E. smithi* as a biological control agent by attracting or guiding them to finding *A. spiniferus* via the parasitoid attractants?”.

Plant synomones are cues that attract parasitic wasps. During the field study, a series of behaviors of *E. smithi* can be observed. They include searching, tapping antennae, and stabbing ovipositors into whitefly individuals on the back of tea leaves. Our research group found that the odors from whitefly-pierced tea leaves (WPTLs), as well as adjacent intact tea leaves (AITLs), strongly attracted the parasitic wasp *E. smithi*. But the odor from far intact tea leaves (FITLs) did not attract the wasp. It is speculated that the whitefly piercing induces WPTLs to emit synomones. Then the synomonal compounds acted as signal molecules that induce defense system of AITLs. In turn, AITLs emitted synomones, too. Previously, our research group found that tea buds, leaves, and tender stems, being pierced and sucked by stylets of tea green leafhoppers, *Empoasca flavescens* F. (Hemiptera: Cicadellidae) and stabbed by ovipositors of the leafhoppers, emitted a detectable level of *trans*-2-hexenal and methyl salicylate (MeSA). These two compounds strongly attracted mymarids, *Stethynium empoascae* Subba Rao (Hymenoptera: Mymaridae) and *Schizophragma parvula* Ogloblin (Hymenoptera: Mymaridae). They are egg parasitoids and the dominant natural enemies of *E. flavescens* [15]. In addition, larvae of the tea geometrid, *Ectropis obliqua* (Prout) (Lepidoptera: Geometrid), and the ash tea geometrid, *Ectropis grisescens* Warren (Lepidoptera: Geometrid), feed on buds, leaves and branches of tea plants and induced the release of detectable level of *trans*-2-hexenal and MeSA. Furthermore, behavioral responses in both the laboratory bioassays and the field trapping experiments in tea plantations confirmed that these two volatile compounds significantly attracted two dominant larval parasitoids of the two tea geometrids, *Protapanteles immunis* (Haliday) (Hymenoptera: Braconidae) and *Parapanteles hyposidrae* (Wilkinson) (Hymenoptera: Braconidae) [16,17,18,19,20,21]. MeSA and salicylic acid (SA) also significantly attracted *P. hyposidrae* (Wilkinson) [22]. Furthermore, in vitro release of the mixture of MeSA and SA or MeSA alone could induce tea plants to emit much more (*E*,*E*)-*α*-farnesene, with the amount released by the mixture of MeSA and SA being slightly larger [22].

We hypothesize that *A. spiniferus* feeding induces the damaged tea plants to produce a synomone that attracts *E. smithi* and primes neighboring tea plants for self-defense, including the subsequent release of their own synomones. Specifically, we aimed to (i) identify the synomonal components associated with whitefly infestation, (ii) determine their attractiveness to *E. smithi* in laboratory bioassays, (iii) evaluate controlled-release formulation of the synomone-based attractants in tea plantations, and (iv) estimate the effect of the formulation on increasing the parasitism rate.

## 2. Results

### 2.1. Odors from Whitefly-Pierced Tea Leaves (WPTL) and Adjacent Intact Tea Leaves (AITL) Significantly Attracted E. smithi in a Y-Tube Olfactometer

**Figure 1:**
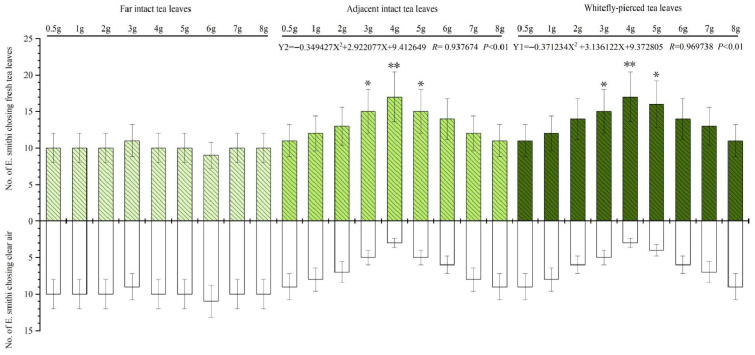
Number of *Encarsia smithi* attracted by each of the three botanical odor sources vs. clear air in a Y-tube olfactometer bioassay. * and ** indicate the difference between the number of wasps choosing each of three botanical odor sources and the number of wasps choosing clear air reaches significance levels of *p* < 0.05 and *p* < 0.01, respectively. Y1 or Y2 = number of wasps, X = tested leaf weight (g).

Compared with CK (clear air), the odors from both WPTLs and AITLs strongly attracted *E. smithi*, but the odor of FITLs hardly attracted any wasps (Figure 1). When weight of WPTLs (or AITLs) was 3 g, 4 g or 5 g, the difference between the number of wasps to choose WPTLs (or AITLs) and the number of wasps to choose CK reached a significant level of *p* < 0.05 (Figure 1).

The number of wasps (Y) choosing odor sources (WPTLs or AITLs) was significantly correlated with the dosage (weight of test leaves) (X) of the two odor sources (0.5 g ≤ X ≤ 8.0 g). Considering the number of wasps selecting WPSTLs as Y1, and selecting AITLs as Y2, with the tested leaf weight (X) measured at 0.5, 1.0, 2.0, 3.0, 4.0, 5.0, 6.0, 7.0, and 8.0 g, the regression equations conform to the quadratic model as follows. When X is at 4 g, the WPTL or AITL displays the maximum attraction. If X > 4 g, the attractiveness decreases, and the high leaf mass may cause saturation or repellence effect.

Y1 = −0.371234 X^2^ + 3.136122 X + 9.372805  *R* = 0.969738, *p* < 0.01

Y2 = −0.349427 X^2^ + 2.922077 X + 9.412649  *R* = 0.937674, *p* < 0.01

### 2.2. A Significant Difference Exists Among the Composition and Content of Volatiles from the Three Types of Fresh Tea Leaves

**Figure 2:**
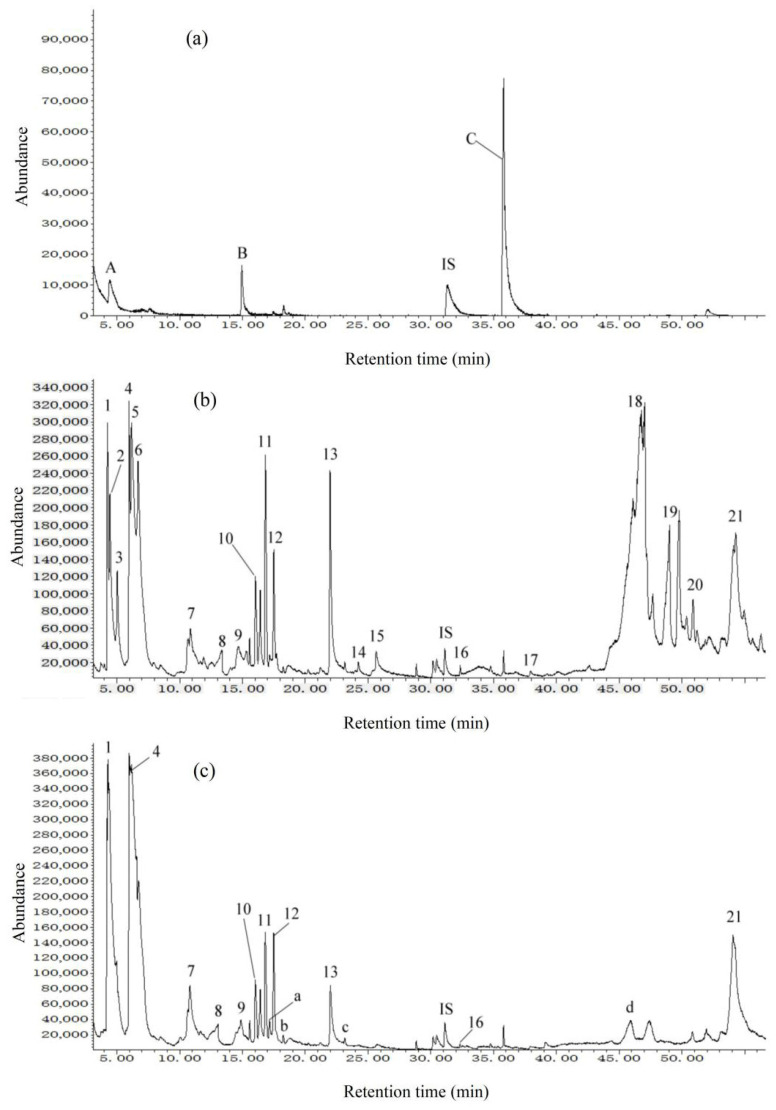
Total ion flow chromatograms of volatiles from far intact tea leaves (**a**), whitefly-pierced tea leaves (**b**), and adjacent intact tea leaves (**c**). The codes of the volatile peaks in Figure 2 are consistent with those in Table 1.

A total of three components were identified from the volatiles of FITL via GC–MS, including A—hexanal, B—ocimene and C—(*E*,*E*)-*α*-farnesene. The number and amount of volatile components from FITLs was much lower than those from the other leaf samples (Figure 2a; Table 1). The peak serial No. in Figure 2 is the same as component serial No. in Table 1.

A total of 21 components was identified from the volatiles of WPTL, including the following components: 1—*cis*-2-hexenal, 2—*cis*-2-hexen-1-ol, 3—*cis*-2-hexen-1-ol, 4—*trans*-2-hexenal, 5—*cis*-3-hexen-1-ol, 6—*cis*-3-hexenyl acetate, 7—benzaldehyde, 8—hexanoic acid, 9—benzyl alcohol, 10—*cis*-linaloloxide, 11—*trans*-linalool oxide (furanoid), 12—geraniol, 13—MeSA, 14—*cis*-3,7-Dimethyl-2,6-octadienal, 15—*trans*-citral, 16—*α*-Ionone, 17—nerolidol, 18—(Z,Z,Z)-9,12,15-octadecatrienoicacid, 19—octadecanoicacid, 20—hexadecane, and 21—*cis*-9-Octadecenamide. Both the number and content of volatile components from WPTLs was significantly higher than those of the FITLs. Of which, *trans*-2-hexenal and MeSA were two of the major volatile components from the WPTL samples (Figure 2b, Table 1).

A total of 15 components were identified from the volatiles of WPTL, including the following components: 1—*cis*-2-hexenal, 4—*trans*-2-hexenal, 7—benzaldehyde, 8—hexanoic acid, 9—benzyl alcohol, 10—*cis*-linaloloxide, 11—*trans*-linalool oxide (furanoid), a—linalool, 12—geraniol, b—*trans*-farnesol, 13—MeSA, c—*β*-cyclocitral, 16—*α*-ionone, d—hexacosane, and 21—*cis*-9-octadecenamide. Of these, *trans*-2-hexenal and MeSA were also the major volatile components (Figure 2c, Table 1).

The detected content of *trans*-2-hexenal from AITLs was 5.0 times of that from WPTLs. This pattern is consistent across three replicates. On 6 April 2022, the tea plants were inoculated with 1-day-old adults of citrus spiny whiteflies and from where the WPTL samples were taken. And on 22 August 2022, AITLs were plucked. WPTLs continuously released *trans*-2-hexenal and MeSA to induce AITLs to produce and emit more *trans*-2-hexenal.

### 2.3. trans-2-Hexenal and MeSA Significantly Attracted Encarsia smithi in a Four-Arm Olfactometer

**Figure 3:**
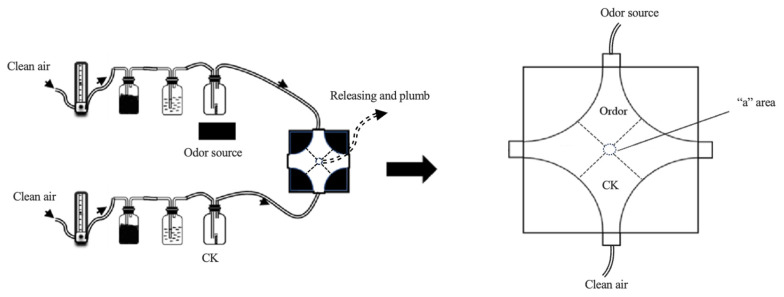
A schematic diagram of the four-arm olfactometer used to test the behavioral responses of *Encarsia smithi* to tea plant synomonal components.

**Figure 4:**
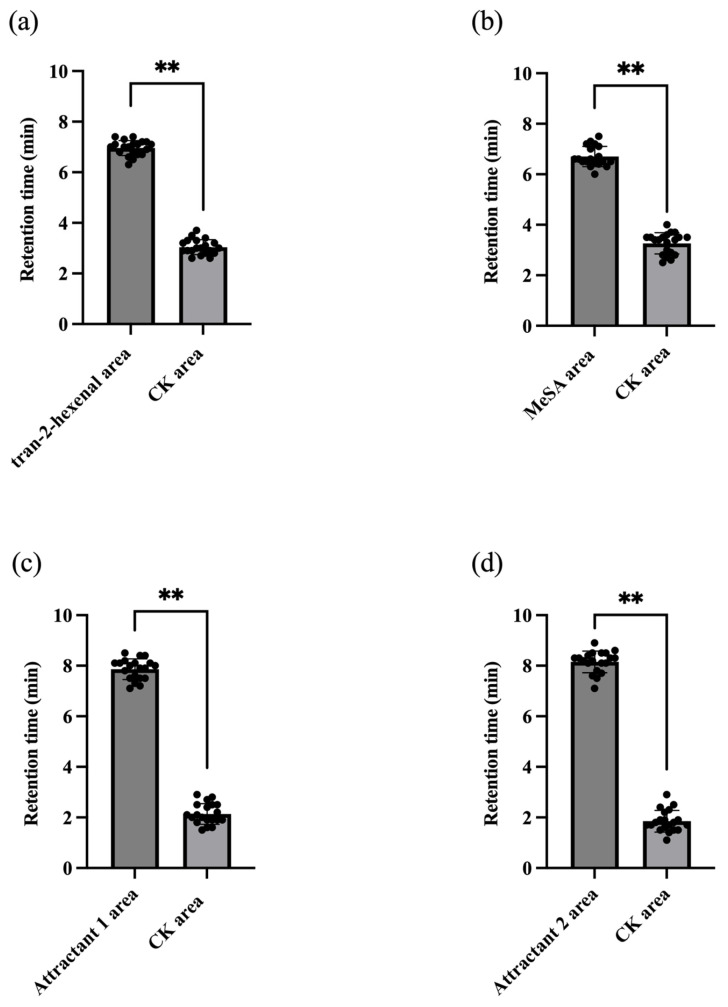
Retention time of *E. smithi* staying in odor field or CK (clear air) area in a four-arm olfactometer (note: paired *t*-test is used; ** indicates the difference reaches a very significant level of *p* < 0.01; Bioassay: (**a**): *trans*-2-hexenal vs. CK (clear air); (**b**): MeSA vs. CK; (**c**): Attractant 1 vs. CK; (**d**): Attractant 2 vs. CK; Each black dot (•) represents the retention time).

The four-arm olfactometer is shown in Figure 3. The average of residence/retention times of the total 20 parasitic wasps tested one by one within the *trans*-2-hexenal odor field (6.96 ± 0.30 min) was significantly higher than within the CK field (3.04 ± 0.30 min) (paired *t*-test, *p* < 0.05) (Figure 4a).

The average of residence/retention times of the total 20 parasitic wasps tested one by one within the MeSA odor field (6.74 ± 0.42 min) was also significantly higher than within the CK field (3.27 ± 0.42 min) (paired *t*-test, *p* < 0.05) (Figure 4b).

The average of residence/retention times of the total 20 parasitic wasps tested one by one within attractant 1 (a blend of *trans*-2-hexenal and MeSA at 1:1 ratio; 7.86 ± 0.41 min) and attractant 2 (a blend of *trans*-2-hexenal and MeSA at a 3:1 ratio; 8.15 ± 0.43 min) odor fields were both significantly higher than within their corresponding CK fields (2.14 ± 0.41 min, and 1.85 ± 0.43 min, respectively) (paired *t*-test, *p* < 0.05) (Figure 4c,d).

These results showed that *trans*-2-hexenal, MeSA, attractant 1 and attractant 2 displayed strong attraction to *E. smithi*, with attractant 2 displaying the strongest attraction.

### 2.4. Attractant 2 Significantly Attracted E. smithi in the Field Trapping Bioassay in Tea Plantations

The nymphal stages of generations 1, 2, 3, and 4 of the whitefly occurred, respectively, from mid-April to late June, from late June to early August, from mid-August to mid-October, and from mid-October to next late March. And adult stages of *E. smithi* roughly coincided with these nymphal stages of the whitefly. From 16 to 20 April, 26 to 30 June, 16 to 20 August, and 16 to 20 October, *E. smithi* populations boomed and the wasp individuals were very abundant and active. They searched for the non-parasitized whitefly nymphs and pupae, then laid eggs on them. So, these four durations were referred to as the trapping periods.

The cumulative catches of *E. smithi* on the sticky traps were counted on the last day of each 5-day trapping period.

**Figure 5:**
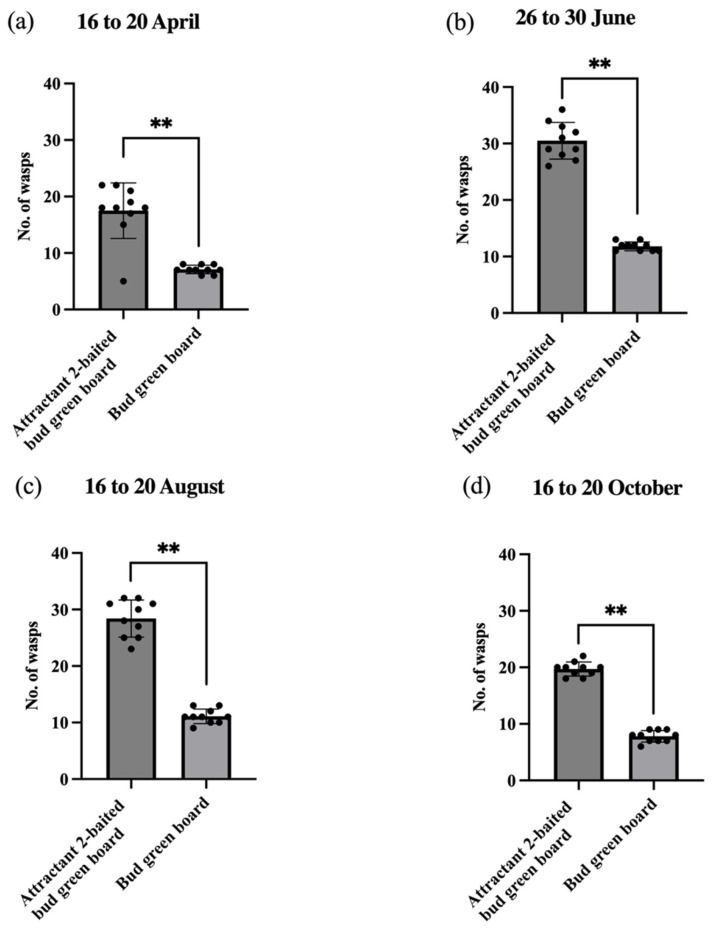
Catches of *E. smithi* on attractant 2-baited bud green boards and the un-baited bud green boards (notes: test site: Jingtingshan Tea Farm of Anhui Province; unpaired *t*-test is used; ** indicates the difference reaches a very significant level of *p* < 0.01; trap duration: (**a**) 16 to 20 April, (**b**) 26 to 30 June, (**c**) 16 to 20 August, (**d**) 16 to 20 October; The each black dot (•) represents the number of wasps captured in every treatment).

The bud green boards baited with attractant 2 lures caught significantly more *E. smithi* wasps than the un-baited CK bud green boards did for all the four trapping periods (unpaired *t*-test, *p* < 0.05), indicating that the parasitoid wasps were strongly attractive to the blend of *trans*-2-hexenal and MeSA at a 3:1 ratio (attractant 2) (Figure 5).

### 2.5. Seven Attractants Significantly Attracted E. smithi with the Attractant 2 Being Best in Tea Field

**Figure 6:**
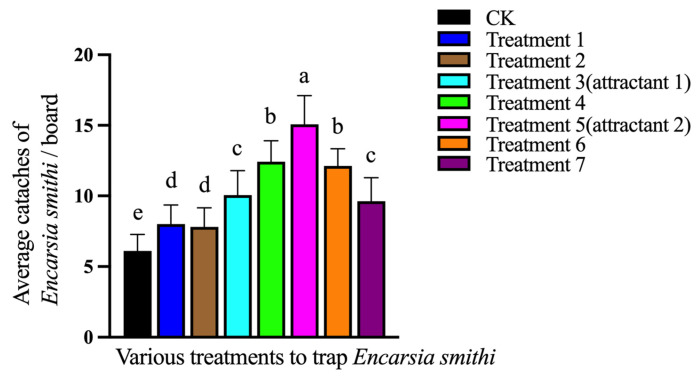
Mean (+SD) numbers of *E. smithi* adults captured on the bud green sticky boards baited with 7 different attractant candidates and the un-baited CK boards. CK: un-baited bud green sticky boards; Treatment 1: 10^−2^ g mL^−1^ *trans*-2-hexenal baited bud green sticky boards; Treatment 2: 10^−2^ g mL^−1^ MeSA baited bud green sticky boards; Treatment 3: attractant 1 baited bud green sticky boards; Treatment 4: blend of *trans*-2-hexenal and MeSA (2:1, *v*/*v*) at 10^−2^ g mL^−1^ baited bud green sticky boards; Treatment 5: attractant 2 baited bud green sticky boards; Treatment 6: blend of *trans*-2-hexenal and MeSA (4:1, *v*/*v*) at 10^−2^ g mL^−1^ baited bud green sticky boards; Treatment 7: blend of *trans*-2-hexenal and MeSA (5:1, *v*/*v*) at 10^−2^ g mL^−1^ baited bud green sticky boards; The difference between columns with different letters (treatments) reaches the significant level (*p* < 0.05).

As shown in Figure 6, CK (bud green sticky boards only) captured a few of *E. smithi* during the 5-day trapping period, and all the attractant-baited bud green sticky boards caught significantly more *E. smithi* than CK did. The attractant 2-baited boards caught the largest number of *E. smithi*, which was significantly more than all other attractant-baited boards including the attractant 1.

### 2.6. Controlled Release of Attractant 2 Could Boost the Whitefly Parasitism Rate by E. smithi

#### 2.6.1. Significantly Promoted the Parasitism Rate by *E. smithi* on the 2nd Generation of Whiteflies

The parasitoid density was initially similar between the tea plantation sections/plots. The continuous release of attractant 2 began on 1 April 2023 in the treated tea plantation plots/sections for enhancing or increasing the parasitic wasps to search for and parasitize the whiteflies within the plots.

The peak period for the 2nd generation of whitefly pupae was 22 July, while August 1 was the peak period of *E. smithi* parasitism on the 2nd generation of whiteflies. On August 1, the parasitism rates (Mean ± SD) in the five attractant 2-treated sections/plots were 12.8 ± 0.6%, 15.7 ± 1.1%, 13.3 ± 1.5%, 14.6 ± 0.3%, and 10.0 ± 1.5%, with an average of 13.3 ± 2.2%, which was 2.9 times of that of the CK sections/plots (Table 2). The difference between these two groups of data reached a significant level (unpaired *t*-test, *t* = 7.2143, *p* < 0.01). The results revealed that attractant 2 could attract the wasps from the surrounding area into the treated plots to significantly improve the parasitism rates.

#### 2.6.2. Significantly Promoted the Parasitism Rate by *E. smithi* on the 3rd Generation of Whiteflies

16 August 2023 was the peak period for the 3rd generation of whitefly pupae, and 26 August 2023 was the peak emergence period of *E. smithi* from these whitefly pupae. On August 26, the parasitism rates in the 5 CK sections/plots were 8.8 ± 0.9%, 8.6 ± 0.9%, 6.2 ± 0.8%, 11.0 ± 2.5% and 8.3 ± 0.5%, with the average being 8.6 ± 1.7%; whereas the parasitism rates in the 5 attractant 2-treated sections/plots were 20.9 ± 1.8%, 21.1 ± 1.5%, 19.0 ± 1.5%, 22.1 ± 0.9% and 24.1 ± 1.7%, with the average being 21.4 ± 1.9%. The average parasitism rate in the attractant 2-treated sections was significantly higher (2.5 times) than that in the CK sections (unpaired *t*-test, *t* = 7.2143, *p* < 0.01) (Table 3).

## 3. Discussion

*Trans*-2-hexenal, MeSA, and salicylic acid (SA) are important synomonal components in tea plants [17]. Tea plants pierced and sucked by the tea green leafhopper, *Empoasca flavescens* F., released abundant *trans*-2-hexenal and MeSA. These two compounds strongly attracted two species of egg parasitoids of *E. flavescens*, mymarids *Stethynium empoascae* Subba Rao and *Schizophragma parvula* Ogloblin [15]. Tea shoots infested with the tea aphid *Toxoptera aurantii* (Boyer) (Hemiptera: Aphididae) also emitted ample *trans*-2-hexenal, which attracted its natural enemies, including the parasitoid *Aphidius* sp. (Hymenoptera: Aphelinidae) and two predators, ladybird *Coccinella septempunctata* L. (Coleoptera: coccinellidae) and lacewing *Chrysopa sinica* Tjeder (Neuroptera: Chrysopidae) [23]. MeSA was strongly attractive to the larval parasitoid *Parapanteles hyposidrae* (Wilkinson) of the ash tea geometrid, *Ectropis grisescens* Warre, and the tea geometrid, *Ectropis obliqua* Prout [18]. The salicylic acid (SA) content in the host plant *Lycium barbarum* L. (Tubiflorae: Solanaceae) significantly increased following infestation by the aphid *Aphis gossypii* Glover (Hemiptera: Aphididae) [24]. MeSA content in tea shoots was significantly increased after exogenous MeSA treatment, piercing and sucking by tea green leafhoppers, or mechanical damage; at the same time, PAL and PPO enzyme activities were also markedly increased to a certain extent. Therefore, MeSA is considered as an important defense-signaling molecule [17].

MeSA motivates and regulates direct and indirect resistances of host plants to pests at the molecular level [25]. Some volatiles released by the leaves of herbivore-infested plants can be perceived by neighboring plants [26]. Green leaf volatiles can induce jasmonate-dependent systemic defense signaling in receiver plants [27]. Herbivore-induced plant volatiles (HIPVs) are known to activate immune signaling in plants, and the potential of selected synthetic HIPVs as sustainable defense priming agents is capable of enhancing citrus immunity by simultaneously activating immune pathways and repressing susceptibility genes [28]. HIPVs not only exert direct toxic effects on insect herbivores but also activate jasmonate-dependent defense pathways in neighboring plants [6,7,8,29].

In the present study, the whitefly adults were introduced to the cultivar ‘Chuyeqi’ tea plants, where they laid eggs that hatched into nymphs. The piercing and sucking by the whitefly nymphs stimulated the defensive reactions of the tea plants infested with the whiteflies. The infested tea plants emit abundant *trans*-2-hexenal and MeSA and change the relative abundances (ratios) of the volatile components to attract natural enemies. Subsequently, *trans*-2-hexenal and MeSA activated the defense system of adjacent intact tea leaves of the nearby tea plants, which released *trans*-2-hexenal and MeSA in turn. This pattern of reaction continued. Through signaling molecules, tea plant individuals “talk” with each other, regulating the resistance of tea plants against the citrus spiny whiteflies [8,25,26,29,30].

Both laboratory bioassays and field trapping experiments verified that *trans*-2-hexenal and MeSA significantly attracted *E. smithi*, with their binary blends exhibiting better attractive efficacy. Field-trapping bioassays on the egg parasitoid attractants for *Stethynium empoascae* [15,31] and the leafhopper attractants for *E. flavescens* [32,33] clearly showed that the blends were superior to that of single or individual component(s). Because the detected emission of *trans*-2-hexenal was distinctly greater than that of MeSA, we prepared attractant 2 (a blend of *trans*-2-hexenal and MeSA) at a ratio of 3:1. The field tests demonstrated that the efficacy of the blend at a 3:1 ratio was significantly better than that at other ratios (1:1, 2:1, 4:1 and 5:1).

Tea plantation habitats are relatively closed and stable, with complex structures of food webs and food chains. In recent years, both organic teas (insecticides are prohibited in tea plantations) and non-pollution teas (the total amount of pesticides used has been restricted in non-pollution plantations) have been greatly expanded in Chinese tea growing regions. Therefore, the use of insecticides has decreased. Hence, *E. smithi*, as well as other natural enemies, has become increasingly prosperous [3]. The slowly released signal molecules (synomonal components), *trans*-2-hexenal and MeSA, mediate “talks” among tea plant individuals to arouse the defensive reaction from proximal to distal [25,26,30,31]. As a result, the control measure could promote a stable parasitism rate by constantly releasing the attractant 2 to regulate *E. smithi* behavior in tea plantations for retaining/maintaining a high population density of *E. smithi*. In Japanese tea plantations, *E. smithi* population has possessed powerful control potential over the *A. spiniferus* populations for many years [10,11,12,13,34,35,36]. In some years, the outbreak of *A. spiniferus* was due to a significant decrease in the parasitism rate [13]. Biological assays have confirmed that organophosphorus, pyrethroid, nereistoxin, and neonicotinoid pesticides, which are used in tea plantations in Japan, are lethal to *E. smithi* [37,38]. The high mortality rates of *E. smithi* due to insecticides might cause the resurgence of the whitefly populations because its sessile nymphs are more protected on the underside of tea leaves [13].

At present, sex pheromones, synomones, kairomones, and other infochemicals are widely used as important semiochemicals-based pest control agents against various pest insects, including aleyrodids [5,39,40,41]. Colored sticky boards with [2,42] or without attractants [43] are currently used for monitoring and controlling adult *A. spiniferus*. To our knowledge, this study is the first to use synomonal components to attract and guide *E. smithi* for enhanced biocontrol against the citrus spiny whitefly.

## 4. Materials and Methods

### 4.1. Attraction of Odors from Whitefly-Pierced Tea Leaves, Adjacent Intact Tea Leaves, and Far Intact Tea Leaves to E. smithi

#### 4.1.1. Tea Leaves

On 6 April 2022, tea plants (13 years old and 1.5 m high) cultivated in pots were selected from the experimental tea garden of China Jiliang University for the study. The tea plant cultivar used was ‘Chuyeqi’. Two pots were placed close together to allow the tea branches to partially interweave. The leaves in the first pot were designated as the “whitefly-pierced tea leaves, i.e., WPTL” and were inoculated with 500 1-day-old adults of citrus spiny whiteflies. The tea bushes were enclosed in a 40-mesh net to prevent the adults from escaping, allowing the adults to freely mate and oviposit on the back of the leaves, occasionally piercing and sucking on tender tea leaves. The cover was not removed until all introduced adults died. The second pot, which was not inoculated with any whiteflies, and the leaves were termed the “adjacent intact tea leaves, i.e., AITL”. The third pot, referred to as the “far intact tea leaves, i.e., FITL”, was placed 30 m away from the other two pot. The experiment was conducted with four replicates for each treatment.

On 6 May 2022, WPTL, AITF, and FITL samples were plucked and used for the administration of incremental dosages in the bioassays of 0.5, 1.0, 2.0, 3.0, 4.0, 5.0, 6.0, 7.0, and 8.0 g. Following the plucking process, the leaves were immediately used as leaf odor sources for the bioassay. CK was clean air.

#### 4.1.2. Insects

From mid-April to mid-June 2022, tea branches containing many pupae of the overwintering generation of whiteflies were collected at intervals from tea plantations in Meijiawu Village, Hangzhou City. Subsequently, the branches were placed in a hydroponic culture in a greenhouse to prompt the emergence of parasitic wasps. In early May, *Encarsia smithi* adults successively emerged from the parasitized pupae of whiteflies. A 60-mesh net was used to cover the branches to prevent wasps from escaping. One-day-old *E. smithi* specimens were collected, kept in 500 mL wide-mouthed bottle sealed the opening with gauze, and supplied with a 10% aqueous honey solution, and allowed to mate freely. The wasps were starved for 15 min before testing. No separation of males and females was conducted for lab bioassays.

#### 4.1.3. Y-Tube Olfactometer and Bioassay Procedures

Following [2], the Y-tube olfactometer consisted of transparent glass, with both the base and each arm being 15 cm long and an internal diameter measuring 1.5 cm. The angle between the two arms was set at 90°. Each arm was connected to either an odor source bottle (containing fresh tea leaves) or a CK bottle (containing clean air), a humidification bottle, an activated carbon filter tube, and a flowmeter. The glass parts were connected using a Teflon tube.

A vacuum pump was used to pull air from the opening of the Y-tube base, and the airflow rate in each arm was set to 80 mL min^−1^. The pumping was performed for 5 min before the test to ensure that the Y-tube system maintained a stable and consistent airflow in both arms. An adult wasp was then introduced through the base opening of the Y-tube using a tube. After entering, the wasp chose either the odor source arm or the CK arm at the Y tube intersection. If it moved 5 cm into the respective arm, the wasp was recorded as having chosen the odor source or CK arm.

The dosage of tested tea leaves during the bioassays was incrementally administered from low to high, specifically 0.5 g, 1.0 g, 2.0 g, 3.0 g, 4.0 g, 5.0 g, 6.0 g, 7.0 g, and 8.0 g. Twenty individual adult wasps were tested against each odor source dosage, one at a time. After every ten individuals were tested, the interior and exterior surfaces of the Y-tube were cleaned with 75% ethanol, and the positions of the odor source and CK arms were shifted to avoid potential positional bias. The same test was repeated four times for each tea leaf dose.

Following the completion of testing each odor source, the Y-tube, odor source bottle, CK bottle, and other glass components were cleaned with potassium dichromate solution and rinsed with distilled water. They were subsequently dried in an oven set to 120 °C before reuse. Then, the activated carbon in the filter was reactivated at 100 °C for 4 h in an oven, then cooled and stored in airtight glass bottles for reuse. The bioassays were performed in a darkroom with a 15 W incandescent light providing lighting 1.5 m above the Y-tube. The test time ranged between 09:00 and 16:00, when the wasps were relatively active. The ambient temperature was at 22–28 °C, the relative humidity was from 65% to 75%, and the light intensity was 3200–3600 lux.

### 4.2. Collection and Identification of Volatiles from WPTLs, AITLs, and FITLs

The tea leaves tested above in “Section 4.1.1” were chosen. On 22 August 2022, during the nymphal stage of the 3rd generation of whitefly, 25 g of WPTLs, AITLs, and FITLs were plucked and each placed in a glass cylinder to collect volatiles, respectively.

The glass cylinder, with a diameter of 10 cm and a volume of 10 L, consisted of two parts that could be tightly connected by fitting the frosted glass together. Filtered clean air was introduced into the air inlet at a flow rate of 100 mL min^−1^, as adjusted using a flow meter. The air outlet was connected to a 150 mg Super Q adsorption column, a flow meter, and a vacuum pump. The flow rate at the inlet was maintained slightly higher than that at the outlet to prevent the ingress of unfiltered ambient air. After 24 h of aeration, the adsorption column was removed and rinsed with 600 μL of HPLC-grade dichloromethane. The eluate was added with 20 μL of 10^−4^ g mL^−1^ decanoic acid ethyl ester as an internal standard, thoroughly mixed, and concentrated to 20 μL under high-purity N_2_ flow, 1 μL of which was injected into a GC-MS system for chemical analyses.

The GC-MS system (GC6890A coupled with MSD6975; Agilent Co., Santa Clara, CA, USA) was equipped with an HP-5MS quartz capillary column (30.0 m × 250 μm × 0.25 μm film thickness). The operational parameters included a splitless injection mode, constant gas flow of 1.0 mL min^−1^, solvent delay of 3 min, injection port temperature of 250 °C, and GC/MS interface temperature of 280 °C. The GC oven was programmed to maintain 50 °C for 5 min, increased to 190 °C at a rate of 3 °C min^−1^, then held at 190 °C for 5 min. GC-MS utilized an electron impact ion source with an ionization energy of 70 eV, performing a full scan at a frequency of 2 Hz and 99.99% helium as the carrier gas.

The compounds were identified by comparing the retention times of the components in the test samples with those of authentic standards, and by comparing the mass spectra with those in the NIST 11 database, as well as in references and the relevant literature [2,15,16]. The relative quantification of each component in the sample was determined based on the ratio of the peak area of each component to that of the internal standard (ethyl decanoate at 10^−4^ g/mL). The experiment was repeated four times for each sample.

### 4.3. Behavioral Bioassay (Response) of E. smithi to trans-2-Hexenal and MeSA

#### 4.3.1. Odor Sources

*trans*-2-hexenal and MeSA (Table 1) were used as odor sources, and their standards were purchased from Sigma Co. with a purity of 98.0%. They were diluted in hexane to make up the following odor sources: ① 10^−4^ g mL^−1^ *trans*-2-hexenal; ② 10^−4^ g mL^−1^ MeSA; ③ attractant 1: blend of 10^−4^ g mL^−1^ *trans*-2-hexenal and 10^−4^ g mL^−1^ MeSA at a ratio of 1:1; ④ attractant 2: blend of 10^−4^ g mL^−1^ *trans*-2-hexenal and 10^−4^ g mL^−1^ MeSA at a ratio of 3:1. Hexane was taken as CK.

#### 4.3.2. Test Insects

The same as “Section 4.1.2”.

#### 4.3.3. Four-Arm Olfactometer Bioassay

As shown in Figure 3, based on the methodologies of [2,44], with slight modifications, a four-arm olfactometer was constructed using clear plexiglass. Each arm was 10 cm long, and the test chamber was 1.5 cm high. The chamber was divided into a central “a” arena and four equal odor fields, each corresponding to one of the four glass inlet arms. Arena “a” served as a mixing zone for the four odor fields, with a diameter of 1 cm. The two opposing arms of the olfactometer were sealed and not utilized in the experiment, while the remaining two arms were designated as the treatment and control (CK) arms. Each arm was connected to an odor source bottle (or CK bottle), a humidification bottle, an activated carbon filter bottle, and a flowmeter with connections made via a Teflon tube. During the experiment, air was drawn through the central entry at the top of the “a” arena using an air pump, and the airflow in each arm was regulated to 100 mL/min.

Before and after each day’s assay, all glassware was cleaned with potassium dichromate solution, rinsed with distilled water, and dried overnight at 100 °C in a hot convection oven. The activated carbon was removed from the filter bottle, reactivated in an oven at 120 °C for 4 h, and then cooled for reuse. The plexiglass components were cleaned with a non-ionic liquid detergent, rinsed with distilled water, followed by 75% ethanol, and air-dried in a clean room. After each bioassay, the interior and exterior of the olfactometer were cleaned with a 75% ethyl alcohol cotton ball, dried, and prepared for subsequent use.

The olfactometer system was housed within a cabinet (1.0 m × 1.0 m × 1.0 m) illuminated from above by diffused uniform lighting from a 15 W fluorescent bulb. The top of the cabinet was connected to the greenhouse, and air was exhausted from the lower part of the cabinet. The cabinet was surrounded by black cloth to minimize external visual stimuli throughout the testing period. The olfactometer was rotated 90° every 5 min to prevent directional bias.

A total of 10 µL of the test odor source was pipetted onto a strip of filter paper. After the hexane evaporated, the strip was inserted into the odor source bottle as the odor source. Simultaneously, 10 µL of hexane was pipetted onto a strip of filter paper, and after the hexane evaporated, the strip was inserted into a CK bottle. A wasp was introduced into the olfactometer through an entry point at the top of arena “a”. The lid was closed, and the airflow system was equilibrated. The wasps were allowed to acclimate for 2 min, after which each replicate was conducted for 10 min. The time spent by the wasps in the odor or CK fields was recorded. Twenty replicates were implemented for each experiment.

The ambient temperature was maintained at 26 ± 1 °C with a relative humidity of 70 ± 5%.

### 4.4. Field Responses of Encarsia smithi to Attractant 2 in Tea Plantations

In eastern China, the nymphal periods of the 1st, 2nd, 3rd, and 4th generations of the whitefly occur from mid-April to late June, late June to early August, mid-August to mid-October, and mid-October to next late March, respectively. The adult stage of each generation of the parasitic wasp, *E. smithi,* coincides with the nymph stage of each generation of the whitefly. *E. smithi* parasitizes nymphs and some pupae of the whitefly.

A block of tea plantation among the mountain forests infested with the whiteflies over the years in Jingtingshan Tea Farm of Anhui Province was chosen, in which four sets of 20 bud green sticky boards, each with a size being 25 cm long × 20 cm wide, were set up at 20 m apart during 16 to 20 April (1st set), 26 to 30 June (2nd set), 16 to 20 August (3rd set), and 16 to 20 October (4th set), 2023, respectively. The “bud green sticky boards” are rectangular cardboards, each with a length of 25 cm and a width of 20 cm. Both of its sides are sticky due to being smeared with sticky, colorless and odorless insect glue. Its color is bud green, because the bud green among 12 types of colors held the strongest attraction to the parasitoid *E. smithi* [43]. Here, we chose the “bud green sticky boards” as the experimental material. The field trials also certified that the “bud green” is strongly attractive to the adults of *Aleurocanthus spiniferus* in tea plantations [2].

During each testing period, 10 sticky boards were randomly chosen as treatment traps, i.e., each was baited with a glass ampoule dispenser (with a 7 mm diameter top opening) that contained 10 mL of attractant 2 solution (blend of 10^−2^ g/mL *trans*-hexenal and 10^−2^ g/mL MeSA in hexane at a volume ratio of 3:1). Simultaneously, the remaining 10 boards, each baited with a glass ampoule dispenser (with a 7 mm diameter top opening) containing 10 mL hexane, were used as un-baited controls. The release rates of the attractant 2 solution or hexane alone from the open ampoules in the field were previously estimated as about 2 mL/day. Thus, the ampoule dispensers can last for the entire 5-day trapping period during each whitefly generation. Each board position was rotated 90° every day to avoid spatial bias.

At the end of the 5-day trapping period, the number of *E. smithi* caught on each trap was counted/recorded.

### 4.5. Comparison of the Attraction of Seven Attractant Candidates to E. smithi

In the morning on 16 October 2025, the large block of organic tea plantation of Jingtingshan Tea Farm of Anhui Province in “Section 4.4” was chosen and randomly divided into 24 sections. They were assigned to 8 treatments with each treatment having three sections (as replicates). The area of each section was 400 m^2^, in which 10 sets of un-baited (CK) bud green sticky boards or 10 sets of attractant-baited bud green sticky boards were set up with a mutual space of 6 m × 6 m. The sections were spaced 10 m apart from each other. The eight treatments were as follows:

CK: Includes 3 sections randomly chosen from 24 sections; in each section, 10 bud green boards, each baited with an open ampoule loaded with 10 mL of hexane, were set up.

Treatment 1: Includes 3 sections randomly chosen from 24 sections; in each section 10 bud green boards, each baited with an open ampoule loaded with 10 mL of 10^−2^ gmL^−1^ *trans*-2-hexenal in hexane solution, were set up.

Treatment 2: Includes 3 sections randomly chosen from 24 sections; in each section 10 bud green boards, each baited with an open ampoule loaded with 10 mL of 10^−2^ gmL^−1^ MeSA in hexane solution, were set up.

Treatment 3: Includes 3 sections randomly chosen from 24 sections; in each section 10 bud green boards, each baited with an open ampoule loaded with 10 mL of attractant 1, i.e., a blend of *trans*-2-hexenal and MeSA (1:1, *v*/*v*) at 10^−2^ g mL^−1^ in hexane solution, were set up.

Treatment 4: Includes 3 sections randomly chosen from 24 sections; in each section 10 bud green boards, each baited with an open ampoule loaded with 10 mL of an attractant, i.e., a blend of *trans*-2-hexenal and MeSA (2:1, *v*/*v*) at 10^−2^ g mL^−1^ in hexane solution, were set up.

Treatment 5: Includes 3 sections randomly chosen from 24 sections; in each section 10 bud green boards, each baited with an open ampoule loaded with 10 mL of attractant 2, i.e., a blend of *trans*-2-hexenal and MeSA (3:1, *v*/*v*) at 10^−2^ g mL^−1^ in hexane solution, were set up.

Treatment 6: Includes 3 sections randomly chosen from 24 sections; in each section 10 bud green boards, each baited with an open ampoule loaded with 10 mL of an attractant, i.e., a blend of *trans*-2-hexenal and MeSA (4:1, *v*/*v*) at 10^−2^ g mL^−1^ in hexane solution, were set up.

Treatment 7: Includes 3 sections randomly chosen from 24 sections; in each section 10 bud green boards, each baited with an open ampoule loaded with 10 mL of an attractant, i.e., a blend of *trans*-2-hexenal and MeSA (5:1, *v*/*v*) at 10^−2^ g mL^−1^ in hexane solution, were set up.

The release rates of the attractant solution or hexane from the open ampoules in the experimental tea plantation were previously estimated as about 2 mL/day. Thus, the ampoule dispensers can last for the entire 5-day trapping period. Each board direction was rotated 90° every day to avoid spatial bias. At the end of the 5-day trapping duration, the numbers of *E. smithi* caught on each trap were recorded.

### 4.6. Controlled Release of Attractant 2 to Enhance the Whitefly Parasitism by E. smithi

On 11 April 2023, a 30 mu (=2 ha; 1 mu = 667 m^2^) organic tea plantation in the mountains of Songyang County, Zhejiang Province was chosen as the experimental field. This tea plantation was severely infested with whiteflies for several years. Within this tea plantation, ten 1 mu square sections with 20 m spacing between two neighboring sections were chosen, of which 5 sections were randomly designated as attractant 2 (controlled release) treatment sections. In each of the five sections, 15 attractant 2 controlled release lures were placed within the tea bushes at 7–8 m apart. Each controlled release lure was made of a 0.1 mm thick PVC bag sealed a felt (4.0 × 2.0 × 0.5 cm) containing 60 mg *trans*-hexenal, 20 mg MeSA, and 200 µL hexane, plus 1 mg butylated hydroxytoluene (BHT) as a stabilizer. The remaining five sections were used as CK plots. In each CK plot, 15 CK PVC-bag dispensers each containing 200 μL of n-hexane plus 1 mg BHT were placed in the tea bushes in the same manner as in the treatment plots.

The attractant 2 lures and the CK dispensers in each section were refreshed on 1 May, 1 June, 1 July, and 1 August, 2023, respectively. According to the field investigation on the developmental progress of the whitefly, the second and third generations of whitefly pupae peaked on 22 July and 16 August, respectively. Moreover, the time-lag effect between the whitefly populations and the *E. smithi* populations was approximately 10 days. Therefore, the number of adults of *E. smithi* peaked on 1 August and 26 August. On these two days, we assessed the parasitism rates of the second- and third-generations of whitefly pupae by *E. smithi*, respectively. The “five-row sampling method” was employed, i.e., sampling five 10 m tea bush rows in each section, and approximately 500 pupae from each sample row were randomly collected. After a wasp emerged from the whitefly pupa, a small hole was left on the backside of the pupa. Pupae with small holes on their backs were identified as being parasitized. The number of live and parasitized pupae was recorded, and the parasitism rate was calculated for each section.

### 4.7. Statistics

All data were checked for normality (Shapiro–Wilk test) and homogeneity of variances (Levene’s test) before further analysis. The data from the Y-tube olfactometer assays, as detailed in Section 4.1.3, were analyzed using a *χ*^2^ test. Data from the four-arm olfactometer assays, as described in Section 4.3.3, were evaluated using a paired *t*-test. Field trapping data (Section 4.4) and the parasitism rate data (Section 4.6) were subjected to an unpaired *t*-test. The trap catch data from Section 4.5 with eight treatments during 15 October to 20 in 2025 were analyzed by one-way analysis of variance (*ANOVA*), followed by Tukey’s multiple comparison test (at *α* = 0.05). Statistical analyses were conducted using the DPS data-processing system [45].

## Figures and Tables

**Table 1:** Qualitative and quantitative analysis on volatile compounds released from far intact tea leaves and whitefly-pierced tea leaves, and adjacent intact tea leaves.

RT (min)	Serial No.			Volatile	Relative Content to Internal Standard (%)		
	WPTLs	AITLs	FITLs		WPTLs	AITLs	FITLs
4.243	1	1		*cis*-2-Hexenal	721.093	2119.565	
4.437	2			*cis*-2-Hexen-1-ol	1020.170		
4.460			A	Hexanal			59.310
5.027	3			1-Hexanol	536.482		
5.965	4	4		*trans*-2-Hexenal	726.402	3642.770	
6.154	5			*cis*-3-Hexen-1-ol	2048.965		
6.697	6			*cis*-3-Hexenyl acetate	2158.277		
10.663	7	7		Benzaldehyde	72.612	94.318	
13.352	8	8		Hexanoic acid	163.487	8.810	
14.674	9	9		Benzyl alcohol	159.194	117.465	
14.937			B	Ocimene			60.918
16.030	10	10		*cis*-Linaloloxide	306.000	185.248	
16.825	11	11		*trans*-Linalool oxide (furanoid)	676.532	283.688	
17.151		a		Linalool		42.361	
17.489	12	12		Geraniol	294.499	261.995	
18.244		b		*trans*-Farnesol		18.548	
21.975	13	13		MeSA	825.815	250.608	
23.148		c		*β*-cyclocitral		10.024	
24.229	14			*cis*-3,7-Dimethyl-2,6-octadienal	43.294		
25.642	15			*trans*-Citral	120.387		
31.107	IS	IS	IS	Ethyl decanoate	100.000	100.000	100.000
32.337	16	16		*α*-Ionone	19.468	2.569	
35.731			C	*α*-Farnesene			62.846
37.951	17			Nerolidol	11.225		
45.927		d		Hexacosane		229.628	
46.694	18			(Z,Z,Z)-9,12,15-octadecatrienoic acid	293.882		
49.017	19			octadecanoic acid	867.809		
50.865	20			Hexadecane	170.946		
54.270	21	21		*cis*-9-Octadecenamide	1159.309	903.118	

**Table 2:** Parasitism rates of *E. smithi* on the pupae of the 2nd generation of *A. spiniferus* in both the controlled-release attractant 2-treated sections and the CK section.

Plot	Controlled-Releasing Attractant 2 Section				CK Section			
	No. of Investigated Pupae	No. of Parasitized Pupae	Parasitism Rate (%)	Parasitism Rate in Section (%)	No. of Investigated Pupae	No. of Parasitized Pupae	Parasitism Rate (%)	Parasitism Rate in Section (%)
1	502	65	12.9	12.8 ± 0.6	486	23	4.7	4.5 ± 0.5
2	498	68	13.7		499	24	4.8	
3	504	61	12.1		502	21	4.2	
4	506	66	13.0		508	19	3.7	
5	488	61	12.5		509	25	4.9	
6	491	77	15.7	15.7 ± 1.1	500	23	4.6	4.7 ± 0.2
7	490	81	16.5		490	23	4.7	
8	492	72	14.6		493	21	4.3	
9	505	86	17.0		503	24	4.8	
10	506	74	14.6		513	25	4.9	
11	504	74	14.7	13.3 ± 1.5	500	36	7.2	6.9 ± 1.2
12	510	60	11.8		499	39	7.8	
13	490	68	13.9		502	40	8.0	
14	486	70	14.4		501	33	6.6	
15	485	56	11.5		506	25	4.9	
16	488	71	14.5	14.6 ± 0.3	511	19	3.7	4.4 ± 0.5
17	486	69	14.2		492	21	4.3	
18	490	72	14.7		493	23	4.7	
19	491	71	14.5		505	24	4.8	
20	505	75	14.9		512	24	4.7	
21	500	50	10.0	10.0 ± 1.5	494	11	2.2	2.3 ± 0.3
22	511	63	12.3		503	10	2.0	
23	483	41	8.5		489	13	2.7	
24	492	52	10.6		511	13	2.5	
25	486	43	8.8		480	10	2.2	

Note: Test site: Songyang County of Zhejiang Province; Investigation time: 1 August 2023.

**Table 3:** Parasitism rates of *E. smithi* on the pupae of the 3rd generation of *A. spiniferus* in both the controlled-release attractant 2-treated sections and CK sections.

Plot	Controlled-Releasing Attractant 2 Section				CK Section			
	No. of Investigated Pupae	No. of Parasitized Pupae	Parasitism Rate (%)	Parasitism Rate in Section (%)	No. of Investigated Pupae	No. of Parasitized Pupae	Parasitism Rate (%)	Parasitism Rate in Section (%)
1	511	108	21.1	20.9 ± 1.8	501	44	8.8	8.8 ± 0.9
2	512	99	19.3		505	47	9.3	
3	500	111	22.2		489	38	7.8	
4	498	94	18.9		487	39	8.0	
5	496	115	23.2		504	50	9.9	
6	485	110	22.7	21.1 ± 1.5	488	41	8.4	8.6 ± 0.9
7	487	93	19.1		500	45	9.0	
8	489	103	21.1		492	41	8.3	
9	488	29	20.0		493	37	7.5	
10	500	112	22.4		496	49	9.9	
11	501	95	19.0	19.0 ± 1.5	506	31	6.1	6.2 ± 0.8
12	515	109	21.2		490	28	5.7	
13	511	96	18.8		488	35	7.2	
14	501	85	17.0		487	33	6.8	
15	505	97	19.2		486	25	5.1	
16	506	117	23.1	22.1 ± 0.9	489	45	11.2	11.0 ± 2.5
17	500	111	22.2		490	44	9.0	
18	502	105	20.9		495	44	14.5	
19	502	114	22.7		496	45	8.3	
20	503	108	21.5		499	43	12.0	
21	504	121	24.0	24.1 ± 1.7	502	41	8.2	8.3 ± 0.5
22	512	135	26.4		503	44	8.7	
23	500	113	22.6		489	44	9.0	
24	594	149	25.1		498	38	7.6	
25	501	111	22.2		483	39	8.1	

Note: test site: Songyang county in Zhejiang Province; Investigation time: 26 August 2023.

## Data Availability

The original contributions presented in this study are included in the article; further inquiries can be directed to the corresponding author.
